# Gastrointestinal pathogens in paediatric patients with diarrhoea during the COVID-19 pandemic in Spain: a multicentre molecular-based prospective study

**DOI:** 10.1007/s00431-026-07213-w

**Published:** 2026-07-01

**Authors:** Alejandro Dashti, Carolina Hernández-Castro, Jin-Min Yuan, Sooria Balasegaram, Pamela Carolina Köster, Begoña Bailo, María Antonia Remacha, Rocío Martínez-Ruiz, Begoña Nogueira, Eva Ramírez de Arellano, Andrea López, María Teresa Llorente, Lourdes Castro-Companioni, Nerea García-Ibáñez, María Dolores Fernández-García, David Carmena, Sergio Sánchez

**Affiliations:** 1 Parasitology Reference and Research Laboratory, National Centre for Microbiology, Health Institute Carlos III, 28820 Majadahonda, Spain; 2 Parasitology Group, Faculty of Medicine, Academic Corporation for the Study of Tropical Pathologies, University of Antioquia, Medellín, Colombia; 3 Field Service, UK Health Security Agency, London, UK; 4 Faculty of Health Sciences, Alfonso X El Sabio University (UAX), Villanueva de La Cañada, Spain; 5 University Health Care Complex of León, León, Spain; 6 Hierro Majadahonda University Hospital, Majadahonda, Spain; 7 Río Hortega University Hospital, Valladolid, Spain; 8 Resistance to Antibiotics and Infections Related to Healthcare Reference and Research Laboratory, National Centre for Microbiology, Health Institute Carlos III, Majadahonda, Spain; 9 Center for Biomedical Research Network in Infectious Diseases (CIBERINFEC), Health Institute Carlos III, Madrid, Spain; 10 National Centre for Microbiology, Health Institute Carlos III, Majadahonda, Spain; 11 Special Pathogens Reference and Research Laboratory, National Centre for Microbiology, Health Institute Carlos III, Majadahonda, Spain; 12 Enterovirus and Viral Gastroenteritis Unit, National Centre for Microbiology, Health Institute Carlos III, Majadahonda, Spain; 13 Center for Biomedical Research Network in Epidemiology and Public Health (CIBERESP), Health Institute Carlos III, Madrid, Spain

**Keywords:** Gastroenteritis, Children, Gastrointestinal pathogens, Molecular detection, Genotyping, Risk factors

## Abstract

Gastroenteritis is a major cause of morbidity in young children worldwide. A prospective multicentre study was conducted from September 2020 to September 2021 in three tertiary hospitals in Spain to assess the burden of the gastrointestinal pathogens causing paediatric gastroenteritis in different Spanish regions, to understand the impact of COVID-19 pandemic in their incidence, and to assess potential risk factors for infection. Stool samples from children aged 0–59 months with acute or chronic diarrhoea were analysed by PCR to detect and characterise gastrointestinal bacteria, parasites, and viruses. Whole-genome sequencing was carried out on enteroaggregative *Escherichia coli* isolates. Epidemiological and clinical data were analysed using univariable and multivariable logistic regression models. A total of 1114 children were included (median age, 16 months; 57.3% male). The most prevalent bacteria were enteropathogenic *E. coli* (16.0%) and *Campylobacter* spp. (15.0%). *Giardia duodenalis* (9.8%) and *Cryptosporidium parvum* (1.8%) were the most common parasites, while norovirus (5.5%) predominated among viruses. Molecular analyses revealed substantial genetic diversity across all pathogens. *Campylobacter* infection increased with age and was associated with fever and bloody diarrhoea, while norovirus infection decreased with age and was associated with vomiting.

*Conclusion* COVID-19 countermeasures substantially altered the transmission of faecal–orally transmitted gastrointestinal pathogens in children, particularly reducing person-to-person spread of human-adapted pathogens such as *C. hominis* and endemic enteroaggregative *E. coli* subtypes. Zoonotic pathogens were largely unaffected, highlighting the selective impact of non-pharmaceutical interventions on anthroponotic transmission.

**Table:** 

**What is Known:** • Paediatric gastroenteritis is caused by faecal–orally transmitted viral, bacterial, and parasitic agents. • COVID-19 countermeasures contributed to the significant reduction of person-to-person transmitted gastrointestinal pathogens.	
**What is New:** • COVID-19 countermeasures drastically interrupted the person-to-person transmission of human-adapted *Cryptosporidium hominis* and endemic enteroaggregative *Escherichia coli* subtypes in Spanish children. • *Cryptosporidium parvum* and other zoonotically transmitted gastrointestinal pathogens were largely unaffected.

**Supplementary Information:**

The online version contains supplementary material available at 10.1007/s00431-026-07213-w.

## Introduction

Gastroenteritis is a clinical syndrome characterised by diarrhoea and/or vomiting, often accompanied by fever and abdominal pain. It is a major contributor to the global disease burden, primarily affecting children younger than 5 years globally [1, 2]. Gastroenteritis is caused by a variety of gastrointestinal (GI) pathogens of viral, bacterial, and parasitic nature [2]. The condition is self-limiting and short-lived (7–14 days) in immunocompetent individuals, but persistent (2–4 weeks) or chronic (> 4 weeks) diarrhoea may occur, with a particular impact on immunocompromised patients. Transmission of pathogens causing gastroenteritis is usually person-to-person, animal-to-person, or via ingestion of food or water contaminated with faecal matter.

In January 2020, the World Health Organization declared severe acute respiratory syndrome coronavirus 2 (SARS-CoV-2) infection a public health emergency, considering it as a pandemic on March 12th 2020. Governments globally were quick to mandate unprecedented measures to reduce the spread and transmission of the virus [3]. In Spain, these included curfew, social distancing, closure of schools and day care centres, use of masks, reinforced hand hygiene, as well as a 98-day nationwide lockdown from March 14th to June 21st 2020. The implementation of these measures not only contributed to the effective mitigation of SARS-CoV-2 infections and other respiratory agents, but also to the significant reduction of faecal–orally transmitted GI pathogens, as reported in various studies worldwide [4–9]. This effect has been observed specifically in paediatric infections in general [10], and particularly in childhood infections caused by GI pathogens [11–13].

In Spain, infections with some of the most common GI pathogens (*Campylobacter jejuni*/*coli*, *Salmonella* spp., *Shigella* spp., *Yersinia enterocolitica*, *Vibrio cholerae*, Shiga toxin-producing *Escherichia coli* (STEC), *Giardia duodenalis*, and *Cryptosporidium* spp.) are considered notifiable diseases. However, limited national surveillance prevents monitoring the true burden of GI pathogens causing gastroenteritis in the country. Information on the impact of the coronavirus disease of 2019 (COVID-19) pandemic on GI pathogen transmission in Spain is restricted to a single-hospital study in Madrid, which reported decreased post-pandemic infection rates for several viral (adenovirus, norovirus, sapovirus) [14], bacterial [*Shigella* spp./enteroinvasive *E. coli* (EIEC)] [15], and parasitic (*Cryptosporidium* spp., *Giardia duodenalis*) [16] agents compared with the pre-pandemic period.

This study aims at (i) obtaining a rough picture of the burden of the most common GI pathogens causing paediatric gastroenteritis in Spain, (ii) expanding our understanding of the impact and consequences of COVID-19 pandemic on their incidence in different Spanish regions, and (iii) assessing the association of potential sociodemographic and clinical risk factors with infections by GI pathogens.

## Methods

### Study design and sample collection

This prospective multicentre study was conducted from September 2020 to September 2021 in three public tertiary hospitals located in the provinces of Madrid (central Spain), Valladolid (central-western Spain), and León (north-western Spain). Participating microbiology laboratories submitted unduplicated fresh stool samples from paediatric patients (0–59 months) with acute (≥ 3 liquid or semi-liquid stools in 24 h, or at least one with mucus, blood, or pus for up to 2 weeks) or chronic (> 4 weeks duration with decreased consistency and increased frequency) diarrhoea. Cases were recruited from emergency departments and inpatient/outpatient clinics. Clinical information regarding the presence of GI symptoms other than diarrhoea was obtained from each sample. A nonprobability convenience sampling strategy was followed, making the estimation of sample size unnecessary. This sampling strategy enabled a total of 1114 patients to be included in the study. Collected stool samples were submitted weekly to the National Centre for Microbiology (NCM, Majadahonda, Spain) and stored at 4 °C (1–5 days) before processing.

### Nucleic acid extraction and purification

Genomic DNA and viral RNA were extracted from the clinical specimens using the QIAamp Fast DNA Stool Mini Kit or the QIAamp Viral RNA Mini Kit (QIAGEN, Hilden, Germany). Nucleic acid extraction was carried out centrally in the Food and Waterborne Bacterial Infections Reference and Research Laboratory. This laboratory processed DNA extracts for bacteria detection, then transferred the remaining DNA to the Parasitology Reference and Research Laboratory for parasite detection and RNA extracts to the Enterovirus and Viral Gastroenteritis Unit for virus detection. Each participating research group applied the diagnostic tools available in their respective laboratories at the time.

### Detection and isolation of diarrheagenic *Escherichia coli*

In parallel to nucleic acid extraction, faecal samples were processed for diarrheagenic *E. coli* (DEC) detection and isolation as described elsewhere [17]. Eight in-house conventional PCR assays were used to amplify the specific genes defining each DEC pathotype. The diagnostic criteria were: STEC, presence of the *stx1*, and/or *stx2*, and/or *stx2f* genes, with or without the *eae* gene; enteroaggregative *E. coli* (EAEC), presence of the *aatA* gene; enteropathogenic *E. coli* (EPEC), presence of the *eae* gene with or without the *bfpA* gene, with absence of *bfpA* indicating atypical EPEC; enterotoxigenic *E. coli* (ETEC), presence of the *eltA* and/or *estA* genes; and EIEC (or *Shigella* spp.), presence of the *ipaH* gene. When culture tested positive for one or more DEC pathotypes, up to 20 individual *E. coli*-like colonies were screened by PCR to obtain isolates, which were biochemically confirmed as *E. coli* by the API 20E system (bioMérieux, Marcy l’Etoile, France) [17].

### Whole-genome sequencing-derived characterisation of EAEC isolates

Molecular characterisation by whole-genome sequencing was restricted to EAEC isolates. Genomic DNA was extracted using the NZY Tissue gDNA Isolation Kit (NZYTech, Lisbon, Portugal). A DNA library was generated using the Nextera DNA Flex Library Preparation Kit (Illumina, San Diego, CA, USA) and sequenced on the Illumina NovaSeq 6000 platform (Illumina). The reads were trimmed and filtered according to quality criteria and analysed for characterisation purposes with an in-house pipeline as previously described [17].

### Molecular detection of other gastrointestinal bacteria

Detection of *Salmonella* spp., *Campylobacter jejuni* and *coli*, *Vibrio* spp., *Yersinia* spp., and *Aeromonas* spp. was conducted by a composite, in-house real-time PCR (RT-PCR) assay adapting a previously published protocol to amplify partial fragments of the *invA*, *cadF*, *toxR*, *lysP*, and aerolysin genes of these bacteria, respectively [18].

### Molecular detection and characterisation of gastrointestinal parasites

The detection and genotyping of GI parasites were carried out by PCR-based methods targeting specific genetic markers: the *ssu* rRNA gene for the detection of *Giardia duodenalis* [19], *Cryptosporidium* spp. [20], and *Entamoeba histolytica*/*dispar* [21, 22]; the *gdh*, *bg*, and *tpi* genes for *G. duodenalis* genotyping [23–26]; the *gp60* gene for *C. parvum* genotyping [27]; the *ssu* rRNA gene for *Blastocystis* detection and subtype (ST) identification [28]; and the ITS region for *Enterocytozoon bieneusi* detection and genotyping [29].

### Molecular detection and characterisation of gastrointestinal viruses

Detection of rotavirus, norovirus (GI and GII genogroups), adenovirus genotypes 40 and 41, and astrovirus was conducted by a multiplex conventional reverse-transcription PCR assay [30]. Norovirus and astrovirus genotypes were determined by direct sequencing of the PCR products. Rotavirus- and adenovirus-positive samples were further genotyped using additional PCR assays targeting the VP7/VP4 and fibre genes, respectively [31, 32].

Detailed information on the PCR/RT-PCR cycling conditions used for the molecular detection and/or characterisation of the investigated pathogens is presented in Table S1. All molecular methods described above are routinely used for diagnostic or research purposes at the NCM.

### Sanger sequencing and phylogenetic analyses

PCR products of the expected size were Sanger sequenced in both directions. Reactions were carried out by capillary electrophoresis using BigDye® Terminator chemistry on an ABI PRISM 3130 Genetic Analyzer (Applied Biosystems). Raw sequences were analysed for species or genotype identification as described elsewhere [33].

### Statistical analysis

Explanatory variables included demographics, symptoms, and co-infections with other GI pathogens. Age was categorised into three groups reflecting children’s eating milestones (0–12, 13–24, and 25–59 months). New variables were created: “diarrhoea only” and two infection variables (“any GI virus” and “any GI virus except norovirus”). Infections with fewer than ten cases were excluded from analyses. The dataset used contained no missing values (Table S2). Univariable analyses calculated odds ratios (ORs) and *P*-values using Chi-squared tests. Multivariable logistic regression models were then built to assess independent associations, including age and hospital setting as a priori confounders. Explanatory variables with *P* < 0.2 or showing > 20% confounding effect were retained. Only variables positively associated with the outcome (OR > 1) were included in the final backward stepwise model. *P*-values were derived from likelihood ratio tests. Analyses were conducted in STATA/SE 15.1.

## Results

### Occurrence of gastrointestinal pathogens

The study population consisted of 1114 children (mean age, 16 months; range, 0–59 months; male 57.3%) originating from Madrid (*n* = 386), Valladolid (*n* = 343), and León (*n* = 385). Table 1 summarises the prevalence of the investigated GI pathogens. Overall, EPEC (16.0%, 178/1.114; 95% CI, 13.9–18.3) and *Campylobacter* spp. (15.0%, 167/1114; 95% CI, 12.9–17.2) were the most prevalent bacterial agents. Other bacteria showed low prevalence rates (< 3%), and *Vibrio* spp. was not detected. Among parasites, *G. duodenalis* was the most frequent (9.8%, 109/1114; 95% CI, 8.1–11.7), followed by *Cryptosporidium* spp. (1.8%, 20/1114; 95% CI, 1.1–2.8), while *Blastocystis* and *E. bieneusi* occurred at < 1%. *E. histolytica*/*E. dispar* were not identified. Among viruses, norovirus (5.5%, 61/1114; 95% CI, 0.4–7.0) and astrovirus (1.3%, 14/1114; 95% CI, 0.7–2.1) predominated, with adenovirus and rotavirus detected in < 1% of samples. Of the 1114 children examined, 530 (47.6%) tested negative for all GI pathogens investigated. Single infections were detected in 427 (38.3%) cases, while 157 (14.1%) involved co-infections (139 with two pathogens, 17 with three, and one with four).

**Table 1:** Prevalence of gastrointestinal pathogens in the surveyed paediatric population (*n* = 1114) according to the hospital centre of origin

GI pathogen	PHMUH (*n* = 386)			RHUH (*n* = 343)			UHCCL (*n* = 385)			All three (*n* = 1.114)		
	**Pos. (*n*)**	**Pos. (%)**	**95% CI**	**Pos. (*n*)**	**Pos. (%)**	**95% CI**	**Pos. (*n*)**	**Pos. (%)**	**95% CI**	**Pos. (*n*)**	**Pos. (%)**	**95% CI**
Bacteria												
*Aeromonas* spp.	5	1.3	0.4–3.0	0	0.0	0.0–1.1^a^	1	0.3	0.0–1.4	6	0.5	0.2–1.2
*Campylobacter* spp.	43	11.1	8.2–14.7	46	13.4	10.0–17.5	78	20.3	16.4–24.6	167	15.0	12.9–17.2
EAEC	8	2.1	0.9–4.0	6	1.8	0.6–3.8	3	0.8	0.2–2.2	17	1.5	0.9–2.4
EIEC/*Shigella* spp.	0	0.0	0.0–1.0^a^	0	0.0	0.0–1.1^a^	1	0.3	0.0–1.4	1	0.1	0.0–0.5
EPEC	57	14.8	11.4–18.7	58	16.9	13.1–21.3	63	16.4	12.8–20.4	178	16.0	13.9–18.3
ETEC	0	0.0	0.0–1.0^a^	1	0.3	0.0–1.6	2	0.5	0.1–1.9	3	0.3	0.1–0.8
STEC	12	3.1	1.6–5.4	4	1.2	0.3–3.0	6	1.6	0.6–3.4	22	2.0	0.1–3.0
*Salmonella* spp.	9	2.3	1.1–4.4	8	2.3	1.0–4.5	8	2.1	0.9–4.1	25	2.2	1.5–3.3
*Vibrio* spp.	0	0.0	0.0–1.0^a^	0	0.0	0.0–1.1^a^	0	0.0	0.0–1.0^a^	0	0.0	0.0–0.3^a^
*Yersinia* spp.	8	2.1	0.9–4.0	4	1.2	0.3–3.0	5	1.3	0.4–3.0	17	1.5	0.9–2.4
Parasites												
*Blastocystis* sp.	7	1.8	0.7–3.7	3	0.9	0.2–3.0	0	0.0	0.0–1.0^a^	10	0.9	0.4–1.6
*Cryptosporidium* spp.	5	1.3	0.4–3.0	4	1.2	0.3–3.0	11	2.9	1.4–5.1	20	1.8	1.1–2.8
*Giardia duodenalis*	48	12.4	9.3–16.2	18	5.3	3.2–8.2	43	11.2	8.2–14.8	109	9.8	8.1–11.7
*Entamoeba histolytica*	0	0.0	0.0–1.0^a^	0	0.0	0.0–1.1^a^	0	0.0	0.0–1.0^a^	0	0.0	0.0–0.3^a^
*Entamoeba dispar*	0	0.0	0.0–1.0^a^	0	0.0	0.0–1.1^a^	0	0.0	0.0–1.0^a^	0	0.0	0.0–0.3^a^
*Enterocytozoon bieneusi*	0	0.0	0.0–1.0^a^	0	0.0	0.0–1.1^a^	2	0.5	0.1–18.6	2	0.2	0.0–0.7
Viruses												
Adenovirus	8	2.1	0.9–4.0	1	0.3	0.0–1.6	1	0.3	0.0–1.4	10	0.9	0.4–1.6
Astrovirus	1	0.3	0.0–1.4	12	3.5	1.8–6.0	1	0.3	0.0–1.4	14	1.3	0.7–2.1
Norovirus GI/GII	29	7.5	5.1–10.6	17	5.0	2.9–7.8	15	3.9	2.2–6.3	61	5.5	0.4–7.0
Rotavirus	1	0.3	0.0–1.4	3	0.9	0.2–2.5	2	0.5	0.1–18.6	6	0.5	0.2–1.2

95% confidence intervals are indicated

*GI* gastrointestinal, *EAEC* Enteroaggregative *Escherichia coli*, *EIEC* Enteroinvasive *Escherichia coli*, *EPEC* Enteropathogenic *Escherichia coli*, *ETEC* Enterotoxigenic *Escherichia coli*, *PHMUH* Puerta de Hierro Majadahonda University Hospital, *RHUH* Río Hortega University Hospital, *STEC* Shiga toxin-producing *Escerichia coli*, *UHCCL* University Health Care Complex of León

^a^One-sided 97.5% confidence interval

Besides diarrhoea, other GI symptoms were reported in 202 (18.1%) cases, including bloody diarrhoea (4.8%), prolonged/chronic diarrhoea (4.0%), fever (3.9%), abdominal pain (2.7%), vomiting and failure to thrive (1.5% each), and reduced appetite (0.3%) (Table S2). Tables 2 and 3 summarise the distribution of symptoms by pathogen. Despite its high prevalence, EPEC infection was mostly characterised by diarrhoea only (87.6%), with chronic diarrhoea and abdominal pain reported in 3.4% of cases each. Bloody diarrhoea occurred in 12.6% of *Campylobacter* and 8.0% of *Salmonella* infections. Among parasites, *Giardia*-positive children had fever (5.5%), abdominal pain, and vomiting (3.7% each), and *Cryptosporidium* infections were occasionally linked to chronic diarrhoea, fever, and vomiting (5.0% each). No symptoms were associated with *Blastocystis* or *E. bieneusi*. Among viruses, vomiting predominated in adenovirus (20.0%) and norovirus (6.6%) infections, whereas astrovirus and rotavirus infection cases only reported diarrhoea.

**Table 2:** Distribution of variables overall and by infection (enteropathogenic *Escherichia coli*, *Campylobacte*r spp., *Giardia duodenalis*, norovirus, *Salmonella* spp.) with measure of association assessed using Chi-squared tests (*n* = 1114)

Variable	Overall		EPEC		*Campylobacter* spp.		*Giardia duodenalis*		Norovirus		*Salmonella* spp.	
	***n***	**%**	***n* (%)**	***P*-value**	***n* (%)**	***P*-value**	***n* (%)**	***P*-value**	***n* (%)**	***P*-value**	***n* (%)**	***P*-value**
Infected	584	52.4	178 (16.0)		167 (15.0)		109 (9.8)		61 (5.5)		25 (2.2)	
Sex												
Female	476	42.7	78 (43.8)	0.75	69 (41.3)	0.69	43 (39.5)	0.47	26 (42.6)	0.99	16 (64.0)	**0.03**
Male	638	57.3	100 (56.2)		98 (58.7)		66 (60.6)		35 (57.4)		9 (36.0)	
Age (months)												
0–12	455	40.8	74 (41.6)	0.69	54 (32.3)	** < 0.001**	36 (33.0)	0.14	27 (44.3)	**0.01**	5 (20.0)	**0.02**
13–24	349	31.3	59 (33.2)		45 (27.0)		35 (32.1)		27 (44.3)		7 (28.0)	
25–59	310	27.8	45 (25.3)		68 (40.7)		38 (34.9)		7 (11.5)		13 (52.0)	
Symptoms^a^												
Diarrhoea only	912	81.9	156 (87.6)	**0.03**	131 (78.4)	0.21	90 (82.6)	0.84	53 (86.9)	0.30	20 (80.0)	0.81
Bloody diarrhoea	53	4.8	5 (2.8)	0.18	21 (12.6)	** < 0.001**	3 (2.8)	0.30	0 (0.0)	0.07	2 (8.0)	0.44
Chronic diarrhoea	45	4.0	6 (3.4)	0.62	2 (1.2)	**0.04**	2 (1.8)	0.22	2 (3.3)	0.76	0 (0.0)	0.30
Fever	43	3.9	3 (1.7)	0.10	12 (7.2)	**0.02**	6 (5.5)	0.35	3 (4.9)	0.66	2 (8.0)	0.28
Abdominal pain	30	2.7	6 (3.4)	0.54	3 (1.8)	0.44	4 (3.7)	0.51	0 (0.0)	0.18	1 (4.0)	0.68
Failure to thrive	17	1.5	3 (1.7)	0.85	0 (0.0)	0.08	1 (0.9)	0.59	0 (0.0)	0.32	0 (0.0)	0.53
Vomiting	17	1.5	1 (0.6)	0.25	1 (0.6)	0.29	4 (3.7)	0.06	4 (6.6)	**0.001**	0 (0.0)	0.53
Reduced appetite	3	0.3	0 (0.0)	0.45	0 (0.0)	0.47	0 (0.0)	0.57	0 (0.0)	0.68	0 (0.0)	0.79
Infections^a^												
EPEC	178	16.0			22 (13.2)	0.28	17 (15.6)	0.91	15 (24.6)	0.06	3 (12.0)	0.58
*Campylobacter* spp.	167	15.0	22 (12.4)	0.28			13 (11.9)	0.35	1 (1.6)	0.003	1 (4.0)	0.12
*Giardia duodenalis*	109	9.8	17 (9.6)	0.91	13 (7.8)	0.35			9 (14.8)	0.18	3 (12.0)	0.71
Norovirus	61	5.5	15 (8.4)	0.06	1 (0.6)	**0.003**	9 (8.3)	0.18			1 (4.0)	0.74
*Salmonella* spp.	25	2.2	3 (1.7)	0.58	1 (0.6)	0.12	3 (2.8)	0.71	1 (1.6)	0.74		
STEC	22	2.0	2 (1.1)	0.37	0 (0.0)	0.05	5 (4.6)	**0.04**	1 (1.6)	0.85	0 (0.0)	0.47
*Cryptosporidium* spp.	20	1.8	3 (1.7)	0.90	0 (0.0)	0.06	2 (1.8)	0.97	0 (0.0)	0.28	1 (4.0)	0.40
EAEC	17	1.5	2 (1.1)	0.63	1 (0.6)	0.29	4 (3.7)	0.06	1 (1.6)	0.94	1 (4.0)	0.31
*Yersinia enterocolitica*	17	1.5	1 (0.6)	0.25	1 (0.6)	0.29	2 (1.8)	0.78	0 (0.0)	0.32	0 (0.0)	0.53
Astrovirus	14	1.3	5 (2.8)	**0.04**	0 (0.0)	0.11	2 (1.8)	0.57	0 (0.0)	0.37	0 (0.0)	0.57
*Blastocystis* sp.	10	0.9	1 (0.6)	0.60	2 (1.2)	0.66	0 (0.0)	0.30	0 (0.0)	0.45	1 (4.0)	0.10
Adenovirus	10	0.9	2 (1.1)	0.73	2 (1.2)	0.66	2 (1.8	0.28	0 (0.0)	0.45	0 (0.0)	0.63
Aeromonas	6	0.5	1 (0.6)	0.96	2 (1.2)	0.21	0 (0.0)	0.42	0 (0.0)	0.55	0 (0.0)	0.71
Rotavirus	6	0.5	3 (1.7)	**0.02**	0 (0.0)	0.30	0 (0.0)	0.42	0 (0.0)	0.55	0 (0.0)	0.71

Statistically significant values are in bold emphases

*EAEC* enteroaggregative *Escherichia coli*, *EPEC* enteropathogenic *Escherichia coli*, *STEC* Shiga toxin-producing *Escherichia coli*

^a^Ordered in decreasing number of cases

**Table 3:** Distribution of variables overall and by infection (Shiga toxin-producing *Escherichia coli*, *Cryptosporidium* spp., enteroaggregative *E. coli*, *Yersinia enterocolitica*, astrovirus) with measure of association assessed using Chi-squared tests (*n* = 1114)

Variable	Overall		STEC		*Cryptosporidium* spp.		EAEC		*Yersinia enterocolitica*		Astrovirus	
	***n***	**%**	***n* (%)**	***P*-value**	***n* (%)**	***P*-value**	***n* (%)**	***P*-value**	***n* (%)**	***P*-value**	***n* (%)**	***P*-value**
Infected	584	52.4	22 (2.0)		20 (1.8)		17 (1.5)		17 (1.5)		14 (1.3)	
Sex												
Female	476	42.7	11 (50.0)	0.49	10 (50.0)	0.51	9 (52.9)	0.39	6 (35.3)	0.53	8 (57.1)	0.27
Male	638	57.3	11 (50.0)		10 (50.0)		8 (47.1)		11 (64.7)		6 (42.9)	
Age (months)												
0–12	455	40.8	7 (31.8)	0.60	2 (10.0)	**0.001**	3 (17.7)	0.14	9 (52.9)	0.44	4 (28.6)	0.11
13–24	349	31.3	7 (31.8)		5 (25.0)		7 (41.2)		3 (17.7)		8 (57.1)	
25–59	310	27.8	8 (36.4)		13 (65.0)		7 (41.2)		5 (29.4)		2 (14.3)	
Symptoms^a^												
Diarrhoea only	912	81.9	21 (95.5)	0.10	18 (90.0)	0.34	17 (100)	0.05	16 (94.1)	0.19	14 (100)	0.08
Bloody diarrhoea	53	4.8	0 (0.0)	0.29	0 (0.0)	0.31	0 (0.0)	0.35	0 (0.0)	0.35	0 (0.0)	0.40
Chronic diarrhoea	45	4.0	0 (0.0)	0.33	1 (5.0)	0.83	0 (0.0)	0.39	0 (0.0)	0.39	0 (0.0)	0.44
Fever	43	3.9	0 (0.0)	0.34	1 (5.0)	0.79	0 (0.0)	0.41	1 (5.9)	0.66	0 (0.0)	0.45
Abdominal pain	30	2.7	0 (0.0)	0.43	0 (0.0)	0.45	0 (0.0)	0.49	0 (0.0)	0.49	0 (0.0)	0.53
Failure to thrive	17	1.5	0 (0.0)	0.56	0 (0.0)	0.57	0 (0.0)	0.61	0 (0.0)	0.61	0 (0.0)	0.64
Vomiting	17	1.5	1 (4.6)	0.24	1 (5.0)	0.20	0 (0.0)	0.61	0 (0.0)	0.61	0 (0.0)	0.64
Reduced appetite	3	0.3	0 (0.0)	0.81	0 (0.0)	0.82	0 (0.0)	0.83	0 (0.0)	0.83	0 (0.0)	0.85
Infections^a^												
EPEC	178	16.0	2 (9.1)	0.37	3 (15.0)	0.90	2 (11.8)	0.63	1 (5.9)	0.25	5 (35.7)	0.04
*Campylobacter* spp.	167	15.0	0 (0.0)	0.05	0 (0.0)	0.06	1 (5.9)	0.29	1 (5.9)	0.29	0 (0.0)	0.11
*Giardia duodenalis*	109	9.8	5 (22.7)	**0.04**	2 (10.0)	0.97	4 (23.5)	0.06	2 (11.8)	0.78	2 (14.3)	0.57
Norovirus	61	5.5	1 (4.6)	0.85	0 (0.0)	0.28	1 (5.9)	0.94	0 (0.0)	0.32	0 (0.0)	0.37
*Salmonella* spp.	25	2.2	0 (0.0)	0.47	1 (5.0)	0.40	1 (5.9)	0.31	0 (0.0)	0.53	0 (0.0)	0.57
STEC	22	2.0			0 (0.0)	0.52	0 (0.0)	0.56	2 (11.8)	**0.003**	1 (7.1)	0.16
*Cryptosporidium* spp.	20	1.8	0 (0.0)	0.52			0 (0.0)	0.57	0 (0.0)	0.57	0 (0.0)	0.61
EAEC	17	1.5	0 (0.0)	0.56	0 (0.0)	0.57			0 (0.0)	0.61	0 (0.0)	0.64
*Yersinia enterocolitica*	17	1.5	2 (9.1)	**0.003**	0 (0.0)	0.57	0 (0.0)	0.61			0 (0.0)	0.64
Astrovirus	14	1.3	1 (4.6)	0.16	0 (0.0)	0.61	0 (0.0)	0.64	(0.0)	0.64		
*Blastocystis* sp.	10	0.9	0 (0.0)	0.65	0 (0.0)	0.67	0 (0.0)	0.69	0 (0.0)	0.69	0 (0.0)	0.72
Adenovirus	10	0.9	0 (0.0)	0.65	0 (0.0)	0.67	1 (5.9)	**0.03**	0 (0.0)	0.69	0 (0.0)	0.72
Aeromonas	6	0.5	0 (0.0)	0.73	0 (0.0)	0.74	0 (0.0)	0.76	0 (0.0)	0.76	0 (0.0)	0.78
Rotavirus	6	0.5	0 (0.0)	0.73	0 (0.0)	0.74	0 (0.0)	0.76	0 (0.0)	0.76	0 (0.0)	0.78

Statistically significant values are in bold emphases

*EAEC* enteroaggregative *Escherichia coli*, *EPEC* enteropathogenic *Escherichia coli*, *STEC* Shiga toxin-producing *Escherichia coli*

^a^Ordered in decreasing number of cases

### Molecular characterisation of EAEC

EAEC was isolated from all the 17 positive samples. Nine distinct sequence types (ST) and 12 distinct serogenotypes were found, with ST10 (47.1%, 8/17) and serogenotype O153:H2 (23.5%, 4/17) being the most common (Table 4 and Table S3). The main genetic characteristics of EAEC isolates are summarised in Table S3.

**Table 4:** Molecular characterisation of the gastrointestinal pathogens identified in the clinical paediatric population investigated in the present study

GI pathogen	Species/genotype	Sub-genotype	No. of isolates	Relative frequency (%)
*Giardia duodenalis*	A	AII	2	18.2
	B	BIII	1	9.1
		BIV	8	72.7
*Cryptosporidium* spp.	*C. parvum*	IIaA15G2R1	9	45.0
		IIaA14G2R1	1	5.0
		IIaA16G3R1	1	5.0
		IIaA17G1R1	1	5.0
		IIdA17G2R1	1	5.0
		Unknown	7	35.0
*Blastocystis* sp.	ST1	Allele 4	2	20.0
	ST2	Allele 12	1	10.0
	ST4	Allele 42	6	60.0
		Unknown	1	10.0
*E. bieneusi*	D	–	1	50.0
	HhSpEb1	–	1	50.0
Norovirus	GII	GII.2[P16]	4	6.6
	GII	GII.3[P12]	42	68.9
	GII	GII.3[P30]	1	1.6
	GII	GII.4[P16]	10	16.4
	GII	GII.4[P31]	2	3.3
	GII	GII.12[P16]	1	1.6
	GII	GII.17[P17]	1	1.6
Astrovirus	–	1	13	92.9
	–	3	1	7.1
Adenovirus	–	41	10	100.0
Rotavirus	–	G1P[8]^a^	1	16.7
	–	G2P[4]	3	50.0
	–	G4P[8]	2	33.3
EAEC	ST10	O3:H2	3	17.6
	ST10	O153:H2	4	23.5
	ST10	O176:H33	1	5.9
	ST12	O4:H5	1	5.9
	ST34	O92:H33	1	5.9
	ST34	ONT:H33	1	5.9
	ST38	O153:H30	1	5.9
	ST68	O99:H6	1	5.9
	ST200	O175:H31	1	5.9
	ST278	O8:H19	1	5.9
	ST2178	O55:H10	1	5.9
	ST3570	O86:H27	1	5.9

*GI* gastrointestinal, *EAEC* enteroaggregative *Escherichia coli*

^a^Vaccine-derived

### Molecular characterisation of gastrointestinal parasites

Successful genotyping of *G. duodenalis* at any loci (*gdh*, *bg*, *tpi*) was achieved in 10.1% (11/109) of positive samples. Sanger sequencing analyses revealed the presence of sub-assemblages AII (18.2%, 2/11), BIII (9.1%, 1/11), and BIV (72.7%, 8/11) (Table 4). All 20 *Cryptosporidium*-positive samples were identified as *C. parvum* (Table 4). Genotyping at the *gp60* gene succeeded in 65.0% (13/20) of cases, revealing genotype families IIa (92.3%, 12/13) and IId (7.7%, 1/13). Of the 19 *Blastocystis*-positive samples, 52.6% (10/19) were subtyped as ST1 (20.0%, 2/10), ST2 (10.0%, 1/10), and ST4 (70.0%, 7/10) (Table 4). Two children were infected with *E. bieneusi* genotype D and novel genotype HhSpEb1. Detailed genotyping features of the parasite-derived sequences can be seen in Table S4.

### Molecular characterisation of gastrointestinal viruses

Most astrovirus-positive samples (92.9%, 13/14) corresponded to the classic genotype 1, while one sample was identified as genotype 3 (Table 4). All ten human adenovirus-positive samples were genotyped as genotype 41 (species F) (Table 4). Norovirus genotyping showed GII.3[P12] as the predominant genotype (68.9%, 42/61), followed by GII.4[P16] (16.4%, 10/61), and GII.2[P16] (6.6%, 4/61) (Table 4). Less frequent genotypes included GII.4[P31] (3.3%), and GII.12[P16], GII.17[P17], and GII.3[P30] (1.6% each). Rotavirus genotyping identified G2P[4] (3/6, 50.0%), G4P[8] (2/6, 33.3%), and vaccine-derived G1P[8] (1/6, 16.6%) (Table 4).

### Risk factor analysis: univariable analysis

Results of the univariable analysis, presented in decreasing order of cases for the variables “symptoms” and “infections” are shown in Tables 2 and 3. EPEC was mainly associated with diarrhoea as the only GI manifestation (*P* = 0.03) and co-infection with astrovirus (*P* = 0.04) and rotavirus (*P* = 0.02). *Campylobacter* was more frequent in children aged 25–59 months (*P* < 0.001), particularly those with bloody (*P* < 0.001) or chronic diarrhoea (*P* = 0.04) or fever (*P* = 0.02) and was positively associated with norovirus (*P* = 0.003). *Giardia duodenalis* co-occurred with STEC (*P* = 0.04), while norovirus was more likely in children under 25 months (*P* = 0.01) and those with vomiting (*P* = 0.001). *Salmonella* (*P* = 0.02) and *Cryptosporidium* (*P* = 0.001) infections were also more common among children aged 25–59 months, and adenovirus was associated with EAEC (*P* = 0.03) and STEC (*P* = 0.003).

### Risk factor analysis: multivariable analysis

Results of the multivariable analysis are summarised in Table 5. Among GI bacteria, the odds of *Campylobacter* infection were highest in children aged 25–59 months (aOR = 2.63; 95% CI, 1.74–3.99; *P* < 0.0001). Fever and bloody diarrhoea were independently associated with *Campylobacter* infection. EPEC infection was associated with co-infection by norovirus and rotavirus, with a stronger effect when considering the composite “any GI virus” variable (aOR = 2.11; 95% CI, 1.28–3.48; *P* = 0.003). Having diarrhoea only, without additional symptoms, was also strongly associated with EPEC infection. STEC infection was associated with *Yersinia* infection (aOR = 6.96; 95% CI, 1.45–33.45; *P* = 0.015) and vice versa (aOR = 6.84; 95% CI, 1.43–32.73; *P* = 0.016), but this related to only two cases of co-infection. For *Salmonella*, odds of infection were lower in males (aOR = 0.40; 95% CI, 0.18–0.92; *P* = 0.03), and highest in children aged 25–59 months (aOR = 4.06; 95% CI, 1.41–11.71; *P* = 0.01).

**Table 5:** Multivariable analysis of risk factors for infection with gastrointestinal pathogens among the surveyed clinical paediatric population

Exposure	*Campylobacter* spp.		EPEC		STEC		Norovirus		Any GI virus except norovirus	
	**aOR**^**a**^** (95% CI)**	***P*-value**	**aOR**^**a**^** (95% CI)**	***P*-value**	**aOR**^**a**^** (95% CI)**	***P*-value**	**aOR**^**a**^** (95% CI)**	***P*-value**	**aOR**^**a**^** (95% CI)**	***P*-value**
Age (months)										
0–12 (reference)	–	–	–	–	–	–	–	–	–	–
13–24	1.37 (0.88–2.13)	0.169	–	–	–	–	1.27 (0.72–2.24)	0.416	–	–
25–59	2.63 (1.74–3.99)	< 0.0001	–	–	–	–	0.34 (0.14–0.79)	0.013	–	–
Diarrhoea only	–	–	1.67 (1.03–2.71)	0.037	–	–	–	–	–	–
Bloody diarrhoea	3.96 (2.16–7.25)	< 0.0001	–	–	–	–	–	–	–	–
Fever	2.12 (1.03–4.36)	0.04	–	–	–	–	–	–	–	–
Vomiting	–	–	–	–	–	–	6.47 (1.96–21.41)	0.002	8.12 (1.67–39.57)	0.01
EPEC	–	–	NA	NA	–	–	1.85 (1.00–3.44)	0.05	2.86 (1.29–6.32)	0.01
*Yersinia* spp.	–	–	–	–	6.96 (1.45–33.45)	0.015	–	–	–	–
Norovirus	–	–	–	–	–	–	NA	NA	–	–
Rotavirus	–	–	–	–	–	–	–	–	–	–
Any GI virus	–	–	2.11 (1.28–3.48)	0.003	–	–	–	–	–	–

*aOR* adjusted odds ratio, *95% CI* 95% confidence interval, *NA* not applicable

^a^All models adjusted for hospital setting and age

Among GI viruses, odds of noroviru*s* infection varied by age, being significantly lower in those aged 25–59 months (aOR = 0.34; 95% CI, 0.14–0.79;* P* = 0.013). Vomiting was strongly associated with norovirus infection (aOR = 6.47; 95% CI, 1.96–21.41; *P* = 0.002). When analysing adenovirus, astrovirus, and rotavirus jointly, both vomiting (aOR = 8.12; 95% CI, 1.67–39.57; *P* = 0.01) and EPEC co-infection (aOR = 2.86; 95% CI, 1.29–6.32; *P* = 0.01) were associated with infection.

Among GI parasites, *Cryptosporidium* infection increased significantly with age (*P* = 0.0001). No variables were associated with *G. duodenalis* and *Blastocystis* infections.

## Discussion

We undertook a large prospective study of paediatric diarrhoeal disease during the COVID-19 pandemic to assess the burden of GI pathogens and the impact and consequences of the pandemic on their incidence. Strengths of this study include a large multicentre cohort from three tertiary hospitals in Spain in different regions, molecular detection of 20 GI pathogens, genotyping of positive samples and/or isolates, and comprehensive analyses of risk factors for infection.

Although no previous Spanish studies have specifically investigated paediatric populations using molecular methods to cover major GI bacteria, the post-pandemic prevalences of *Campylobacter* spp., *Salmonella* spp., EIEC/*Shigella* spp., *Yersinia enterocolitica*, and *Aeromonas* spp. infections observed in our study are in line with pre-pandemic findings from all-age gastroenteritis patients [15]. Regarding DEC, the only pre-pandemic Spanish reference is a study of gastroenteritis patients of all ages, but with special attention to children ≤ 5 years old [17]. Our study focussed on the same age group from three of the same hospitals and used the same DEC detection methodology. Comparison of both studies showed a statistically significant post-pandemic decrease only for EAEC (*P* < 0.001) and STEC (*P* = 0.002). This decrease in the EAEC (but not in the STEC) prevalence after the lockdown was also observed in the USA [34]. However, according to our results, this decrease affected EAEC subtypes differently. Specifically, EAEC O126:H27-ST200, O111:H21-ST40, and O92:H33-ST34 are among the most prevalent subtypes in EAEC strains from Spain [17] and other high-income countries [35–40], and even linked to outbreaks of gastroenteritis [41–43]. In a pre-lockdown Spanish study, two presumptive outbreaks of EAEC infection were identified simultaneously in different nursery schools, one caused by EAEC O126:H27-ST200 and the other by O111:H21-ST40, affecting children across different classrooms and educational levels [44]. In our post-pandemic study, EAEC O126:H27-ST200 and O111:H21-ST40, which previously accounted for 18.0% and 17.0%, respectively, of the EAEC isolates [17], were not identified at all. Similarly, EAEC O92:H33-ST34, which previously accounted for 14.0% of EAEC isolates [17], appeared in only one isolate (0.1%) well after the lockdown. These findings suggest that previous strict confinement, mobility restrictions, and social distancing also interrupted the person-to-person transmission of endemic EAEC subtypes (O126:H27-ST200, O111:H21-ST40, and O92:H33-ST34), while leaving other EAEC subtypes and DEC pathotypes (except for STEC), largely unaffected.

Among GI parasites, *G. duodenalis* was detected in 10% of the children, consistent with other PCR-based studies in Spain [45–49]. Our molecular data confirmed the predominance of assemblage B (82%, 9/11) over assemblage A (18%, 2/11), in line with previous Spanish surveys regardless of age group and clinical status [33, 50–52].

*Cryptosporidium* infections were detected in < 2% of children, at the lower end of the 1–15% range reported in Spanish paediatric populations with [45, 46, 53] and without [33, 48, 49] clinical manifestations. Notably, all infections were caused by zoonotic *C. parvum*. This result was puzzling, as anthroponotic *C. hominis* is known to cause three out of four cases of human cryptosporidiosis in Spain [50, 54–57], and this was also the ratio anticipated in this survey. These findings suggest that previous confinement and social distancing disrupted person-to-person *C. hominis* transmission, leaving zoonotic *C. parvum* unaffected. Similar post-pandemic reductions in *C. hominis* transmission have been reported in England and Wales [8, 58], Scotland [59], New Zealand [60], and Sweden [61]. This is the first molecular evidence on this phenomenon reported in Spain to date.

In this study, only 0.4% of children tested positive for *Blastocystis*, far lower than the 3–43% reported in other Spanish paediatric populations [33, 45, 46, 49, 62]. Genotyping showed the absence of ST3, the most common human subtype worldwide [63] and in Spain [62, 64, 65], alongside ST1, ST2, and ST4. Consistent with our findings for *C. hominis* and the endemic EAEC subtypes, we hypothesise that COVID-19 measures likely reduced person-to-person transmission of *Blastocystis* ST3.

As for *E. bieneusi*, this study presents the first reported infections in immunocompetent children in Spain. Previous national reports documented sporadic *E. bieneusi* infection cases in transplant recipients [66], HIV-infected individuals [46, 67, 68], returning travellers [69], and immunocompetent adults [70]. Genotype D, previously reported in non-HIV-infected renal transplant recipients [66], was found in our study, in addition to novel genotype HhSpEb1.

Regarding viruses, both astrovirus and adenovirus genotyping results revealed the predominance of well-known genotypes associated with paediatric gastroenteritis. The vast majority of astrovirus-positive samples corresponded to the classic genotype 1, in agreement with global surveillance data [71]. However, the limited genetic diversity observed in astrovirus (despite its well-documented diversity), may be partially influenced by the primer design used in the multiplex PCR assay, based on a genotype 1 reference strain [30]. Likewise, all ten adenovirus-positive samples were identified as genotype 41, consistently reported as the primary enteric adenovirus linked to paediatric acute gastroenteritis. Genotyping of norovirus-positive samples revealed a predominance of GII.3[P12], followed by GII.4[P16], aligning with prior reports on the distribution of these genotypes in Spain, frequently causing large outbreaks [72–74]. Regarding rotaviruses, our predominant genotypes, G2P[2] and G4P[8], are among the least commonly reported in Spain [75]. This discrepancy may reflect regional variations in genotype circulation, although it could be influenced by shifts in surveillance dynamics during the COVID-19 pandemic.

Our risk factor analyses evidenced several associations between age, clinical features, and infection by bacterial and viral (but not parasitic) GI pathogens. *Campylobacter* infection increased with age and was associated with fever and bloody diarrhoea, in line with findings described in large multicentre surveys. For instance, the Malnutrition and Enteric Disease (MAL-ED) study evidenced that fever and bloody diarrhoea were strong clinical predictors of *Campylobacter* infection, distinguishing it from viral causes of gastroenteritis [76]. The MAL-ED study also demonstrated frequent co-detection of viral pathogens and DEC, providing support for our finding of an association of norovirus and rotavirus with EPEC, as well as the observation that having only diarrhoea, without additional symptoms, is associated with EPEC infection. Similarly, higher odds of *Salmonella* infection in older children have been reported in paediatric cohorts, consistent with dietary diversification and greater environmental exposure [76]. With respect to viral pathogens, norovirus incidence has been shown to fluctuate with age, a pattern attributed to waning maternal immunity and the acquisition of strain-specific immunity [77], and vomiting is a well-established clinical predictor of norovirus infection [78].

Several limitations of this study should be considered when interpreting the results obtained. First, the prevalence, molecular, and risk factor data may not be representative of the national situation, as these parameters can vary across regions, age groups, and clinical statuses. Second, highly sensitive PCR assays may artificially overestimate the apparent prevalence of certain microorganisms, mainly due to asymptomatic carriage or the amplification of genetic material from non-viable microorganisms. Third, the use of a diverse set of molecular methods for detection and genotyping may have influenced the comparative diagnostic performance. Fourth, the absence of pre-COVID-19 pandemic data from this same population may have introduced bias into some of the conclusions drawn.

Our findings indicate that COVID-19 countermeasures substantially affected the transmission dynamics of certain faecal–oral GI pathogens in young children. Strict confinement, reduced mobility, and social distancing appear to have disproportionately interrupted person-to-person transmission of human-adapted pathogens and/or genotypes, as evidenced by the absence or marked decline of *C. hominis* and the endemic subtypes of EAEC. In contrast, zoonotic pathogens or genotypes were largely unaffected, highlighting the selective effect of these interventions on anthroponotic spread. These results underscore how non-pharmaceutical interventions implemented during the COVID-19 pandemic were able to reshape the epidemiology of common GI pathogens.

## Supplementary Information

Below is the link to the electronic supplementary material.

ESM 1 (DOCX 29.0 KB)

ESM 2 (XLSX 119 KB)

ESM 3 (XLSX 14.4 KB)

ESM 4 (DOCX 23.7 KB)

## Data Availability

FASTQ sequences of EAEC isolates were deposited in the National Center for Biotechnology Information Short Read Archive under the BioProject PRJNA1233819. Accession numbers for each sequence are listed in Table S3. The *G. duodenalis*, *C. parvum*, *Blastocystis*, and *E. bieneusi* sequences generated in this study have been deposited in GenBank. Accession number of each sequence are listed in Table S4. Representative viral sequences generated in this study have been deposited in GenBank under accession numbers PZ019914‒PZ019921 (norovirus), PZ019025 and PZ019026 (astrovirus), PZ019027 (adenovirus), and PZ019028‒PZ019035 (rotavirus).

## Abbreviations

COVID-19: Coronavirus disease of 2019
DEC: Diarrheagenic *E. coli*
EAEC: Enteroaggregative *E. coli*
EPEC: Enteropathogenic *E. coli*
GI: Gastrointestinal
STEC: Shiga toxin-producing *E. coli*
