# Supplementary material for: Gastrointestinal pathogens in paediatric patients with diarrhoea during the COVID-19 pandemic in Spain: a multicentre molecular-based prospective study
Source: Eur J Pediatr. 2026 Jul 1;185(7):545. doi: 10.1007/s00431-026-07213-w (PMC13328251; doi:10.1007/s00431-026-07213-w)
Supplement: Supplementary file 1 — (DOCX 29.0 KB) [file 431_2026_7213_MOESM1_ESM.docx]

**Table S1** PCR cycling conditions used for the molecular identification and/or characterization of the gastrointestinal parasitic, bacterial, and viral pathogens investigated in the present study.

|  |  |  | **Temperature and time** | | | |  |  |  |
| --- | --- | --- | --- | --- | --- | --- | --- | --- | --- |
| **Target organism** | **Locus** | **Reverse transcription** | **Initial denaturation** | **Denaturation** | **Annealing** | **Extension** | **No. cycles** | **Final extension** | **Reference** |
| *Giardia duodenalis* | *ssu* rRNA | – | 95°C 15 min | 95°C 15 s | 60°C 1 min | 72°C 30 s | 45 | – | [1] |
|  | *gdh* | – | 95°C 3 min | 95°C 30 s | 55°C 30 s | 72°C 1 min | 35 | 72°C 7 min | [2] |
|  | *bg* | – | 95°C 7 min | 95°C 30 s | 65/55°C 30 s | 72°C 1 min | 35 | 72°C 7 min | [3] |
|  | *tpi* | – | 94°C 5 min | 94°C 45 s | 50°C 45 s | 72°C 1 min | 35 | 72°C 10 min | [4] |
| *Cryptosporidium* spp. | *ssu* rRNA | – | 94°C 3 min | 94°C 40 s | 50°C 40 s | 72°C 1 min | 35 | 72°C 10 min | [5] |
|  | *gp60* | – | 95°C 5 min | 94°C 45 s | 59/50°C 45 s | 72°C 1 min | 35 | 72°C 10 min | [6] |
| *Entamoeba histolytica/dispar* | *ssu* rRNA | – | 95°C 15 min | 95°C 15 s | 60°C 1 min | 72°C 30 s | 45 | – | [7] |
| *Blastocystis* sp. | *ssu* rRNA | – | 95°C 3 min | 94°C 1 min | 59°C 1 min | 72°C 1 min | 30 | 72°C 2 min | [8] |
| *Enterocytozoon bienenusi* | ITS | – | 94°C 3 min | 94°C 30 s | 57°C 30 s | 72°C 40 s | 35 | 72°C 10 min | [9] |
| STEC | *stx1* | – | 94°C 5 min | 94°C 30 s | 56°C 40 s | 72°C 1 min | 25 | 72°C 7 min | [10] |
|  | *stx2* | – | 94°C 5 min | 94°C 30 s | 56°C 40 s | 72°C 1 min | 25 | 72°C 7 min | [10] |
|  | *stx2F* | – | 94°C 5 min | 94°C 30 s | 56°C 40 s | 72°C 1 min | 25 | 72°C 7 min | [10] |
| STEC, EPEC | *eae* | – | 94°C 5 min | 94°C 30 s | 56°C 40 s | 72°C 1 min | 25 | 72°C 7 min | [10] |
| tEPEC | *bfpA* | – | 94°C 5 min | 94°C 30 s | 56°C 40 s | 72°C 1 min | 25 | 72°C 7 min | [10] |
| EAEC | *aatA* | – | 94°C 5 min | 94°C 30 s | 56°C 40 s | 72°C 1 min | 25 | 72°C 7 min | [10] |
| ETEC | *eltA* | – | 94°C 5 min | 94°C 30 s | 56°C 40 s | 72°C 1 min | 25 | 72°C 7 min | [10] |
|  | *estA* | – | 94°C 5 min | 94°C 30 s | 56°C 40 s | 72°C 1 min | 25 | 72°C 7 min | [10] |
| EIEC/*Shigella* spp. | *ipaH* | – | 94°C 5 min | 94°C 30 s | 56°C 40 s | 72°C 1 min | 25 | 72°C 7 min | [10] |
| *Salmonella* spp. | *invA* | – | 95°C 15 min | 95°C 15 s | 60°C 1 min | – | 40 | – | This study |
| *Campylobacter jejuni* and *coli* | *cadF* | – | 95°C 15 min | 95°C 15 s | 60°C 1 min | – | 40 | – | This study |
| *Vibrio* spp. | *toxR* | – | 95°C 15 min | 95°C 15 s | 60°C 1 min | – | 40 | – | This study |
| *Yersinia* spp. | *lysP* | – | 95°C 15 min | 95°C 15 s | 60°C 1 min | – | 40 | – | This study |
| *Aeromonas* spp. | *aer* | – | 95°C 15 min | 95°C 15 s | 60°C 1 min | – | 40 | – | This study |
| Rotavirus | VP6 | 42°C 45 min | 95°C 15 min | 94°C 30 s | 48°C 30 s | 72°C 1 min | 35 | 72°C 7 min | [11] |
|  | VP7 | 50°C 30 min | 95°C 15 min | 95°C 30 s | 50°C 30 s | 72°C 45 s | 35 | 72°C 7 min | [12] |
|  |  | – | 94°C 2 min | 94°C 45 s | 42°C 30 s | 72°C 1 min | 35 | 72°C 7 min |  |
|  | VP4 | 50°C 30 min | 95°C 15 min | 95°C 30 s | 50°C 30 s | 72°C 45 s | 35 | 72°C 7 min | [12] |
|  |  | – | 94°C 2 min | 94°C 45 s | 42°C 30 s | 72°C 1 min | 35 | 72°C 7 min |  |
| Norovirus GI | ORF1-RdRp/ORF1-VP1 | 42°C 45 min | 95°C 15 min | 94°C 30 s | 48°C 30 s | 72°C 1 min | 35 | 72°C 7 min | [11] |
| Norovirus GII | ORF1-RdRp/ORF1-VP1 | 42°C 45 min | 95°C 15 min | 94°C 30 s | 48°C 30 s | 72°C 1 min | 35 | 72°C 7 min | [11] |
| Adenovirus | E1A | 42°C 45 min | 95°C 15 min | 94°C 30 s | 48°C 30 s | 72°C 1 min | 35 | 72°C 7 min | [11] |
|  | Fiber | – | 94°C 5 min | 94°C 45 s | 54°C 45 s | 72°C 1 min | 35 | 72°C 10 min | [13] |
| Astrovirus | ORF1A | 42°C 45 min | 95°C 15 min | 94°C 30 s | 48°C 30 s | 72°C 1 min | 35 | 72°C 7 min | [11] |

*bg*: β-giardin; *gdh*: EAEC: Enteroaggregative *Escherichia coli*; EIEC: Enteroinvasive *Escherichia coli*; EPEC: Enteropathogenic *Escherichia coli*; ETEC: Enterotoxigenic *Escherichia coli*; Glutamate dehydrogenase; ITS: Internal transcribed spacer; *gp60*: 60 kDa glycoprotein; *ssu* rRNA: Small subunit ribosomal RNA; STEC: Shiga toxin-producing *Escherichia coli*; tEPEC: Typical Enteropathogenic *Escherichia coli*; *tpi*: Triose phosphate isomerase.

**References**

1. Verweij JJ, Schinkel J, Laeijendecker D, van Rooyen MA, van Lieshout L, Polderman AM (2003) Real-time PCR for the detection of *Giardia lamblia*. Mol Cell Probes 17:223-225. https://doi.org/10.1016/s0890-8508(03)00057-4

2. Read CM, Monis PT, Thompson RC (2004) Discrimination of all genotypes of *Giardia duodenalis* at the glutamate dehydrogenase locus using PCR-RFLP. Infect Genet Evol 4:125-130. https://doi.org/10.1016/j.meegid.2004.02.001

3. Lalle M, Pozio E, Capelli G, Bruschi F, Crotti D, Caccio SM (2005) Genetic heterogeneity at the beta-giardin locus among human and animal isolates of *Giardia duodenalis* and identification of potentially zoonotic subgenotypes. Int J Parasitol 35:207-213. https://doi.org/10.1016/j.ijpara.2004.10.022

4. Sulaiman IM, Fayer R, Bern C, Gilman RH, Trout JM, Schantz PM, Das P, Lal AA, Xiao L (2003) Triosephosphate isomerase gene characterization and potential zoonotic transmission of *Giardia duodenalis*. Emerg Infect Dis 9:1444-1452. https://doi.org/10.3201/eid0911.030084

5. Tiangtip R, Jongwutiwes S (2002) Molecular analysis of *Cryptosporidium* species isolated from HIV-infected patients in Thailand. Trop Med Int Health 7:357-364. https://doi.org/10.1046/j.1365-3156.2002.00855.x

6. Feltus DC, Giddings CW, Schneck BL, Monson T, Warshauer D, McEvoy JM (2006) Evidence supporting zoonotic transmission of *Cryptosporidium* spp. in Wisconsin. J Clin Microbiol 44:4303-4308. https://doi.org/10.1128/JCM.01067-06

7. Verweij JJ, Oostvogel F, Brienen EA, Nang-Beifubah A, Ziem J, Polderman AM (2003) Prevalence of *Entamoeba histolytica* and *Entamoeba dispar* in northern Ghana. Trop Med Int Health 8:1153-1156. https://doi.org/10.1046/j.1360-2276.2003.01145.x

8. Scicluna SM, Tawari B, Clark CG (2006) DNA barcoding of *Blastocystis*. Protist 157:77-85. https://doi.org/10.1016/j.protis.2005.12.001

9. Buckholt MA, Lee JH, Tzipori S (2002) Prevalence of *Enterocytozoon bieneusi* in swine: an 18-month survey at a slaughterhouse in Massachusetts. Appl Environ Microbiol 68:2595-2599. https://doi.org/10.1128/AEM.68.5.2595-2599.2002

10. Llorente MT, Escudero R, Ramiro R, Remacha MA, Martínez-Ruiz R, Galán-Sánchez F, de Frutos M, Elía M, Onrubia I, Sánchez S (2023) Enteroaggregative *Escherichia coli* as etiological agent of endemic diarrhea in Spain: A prospective multicenter prevalence study with molecular characterization of isolates. Front Microbiol 14:1120285. https://doi.org/10.3389/fmicb.2023.1120285

11. Mitra S, Nayak MK, Majumdar A, Sinha A, Chatterjee S, Deb A, Chawla-Sarkar M, Dutta S (2020) Development and evaluation of a multiplex conventional reverse-transcription polymerase chain reaction assay for detection of common viral pathogens causing acute gastroenteritis. Diagn Microbiol Infect Dis 97:115061. https://doi.org/10.1016/j.diagmicrobio.2020.115061

12. Mijatovic-Rustempasic S, Esona MD, Williams AL, Bowen MD (2016) Sensitive and specific nested PCR assay for detection of rotavirus A in samples with a low viral load. J Virol Methods 236:41-46. https://doi.org/10.1016/j.jviromet.2016.07.007

13. Xu W, McDonough MC, Erdman DD (2000) Species-specific identification of human adenoviruses by a multiplex PCR assay. J Clin Microbiol 38:4114-4120. https://doi.org/10.1128/JCM.38.11.4114-4120.2000
