# Supplementary material for: Gastrointestinal pathogens in paediatric patients with diarrhoea during the COVID-19 pandemic in Spain: a multicentre molecular-based prospective study
Source: Eur J Pediatr. 2026 Jul 1;185(7):545. doi: 10.1007/s00431-026-07213-w (PMC13328251; doi:10.1007/s00431-026-07213-w)
Supplement: Supplementary file 4 — (DOCX 23.7 KB) [file 431_2026_7213_MOESM4_ESM.docx]

**Table S4** Frequency and molecular diversity of the gastrointestinal parasites successfully characterized at the different loci used for detection and genotyping purposes in the present study. GenBank accession numbers are provided.

| **Species** | **Species/ genotype** | **Sub-genotype** | **No. isolates** | **Locus** | **Reference sequence** | **Stretch** | **Single nucleotide polymorphisms** | **GenBank ID** |
| --- | --- | --- | --- | --- | --- | --- | --- | --- |
| *Giardia duodenalis* | A | AII | 2 | *gdh* | L40510 | 64‒447 | None | ON165669 |
|  | B | BIII | 1 | *gdh* | AF069059 | 29‒408 | C309T | ON165670 |
|  | B | BIV | 2 | *gdh* | L40508 | 89‒491 | None | ON165671 |
|  | B | BIV | 5 | *gdh* | L40508 | 88‒496 | T183C, T387C, C396T, C423T | ON165672 |
|  | B | BIV | 1 | *gdh* | L40508 | 76‒496 | C259T | ON165673 |
|  | B | B | 4 | *bg* | AY072727 | 102‒592 | G159A, C165T, C309T, C324T, C393T, T471C | ON165674 |
|  | B | B | 1 | *bg* | AY072727 | 100‒590 | C165T, C309T | ON165675 |
|  |  |  |  |  |  |  |  |  |
|  | B | BIV | 2 | *tpi* | AF069560 | 1‒479 | None | ON165676 |
|  | B | BIV | 1 | *tpi* | AF069560 | 1‒479 | T135Y, T208Y | ON165677 |
|  | B | BIV | 1 | *tpi* | AF069560 | 1‒479 | G295R | ON165678 |
|  | B | BIV | 1 | *tpi* | AF069560 | 1‒479 | A395G | ON165679 |
| *Cryptosporidium* | *C. parvum* | ‒ | 15 | *ssu* rRNA | AF112571 | 530‒1030 | A646G, T649G, 686_689DelTAAT, T693A | ON161760 |
|  | *C. parvum* | ‒ | 2 | *ssu* rRNA | AF112571 | 598‒1030 | A646G, T649G, 686_689DelTAAT | ON161761 |
|  | *C. parvum* | ‒ | 1 | *ssu* rRNA | AF112571 | 633‒998 | A646G, T648_650DelTTA, T663C, 686_689DelTAAT, T820W | ON161762 |
|  | *C. parvum* | ‒ | 1 | *ssu* rRNA | AF112571 | 682‒1026 | 686_689DelTAAT, T693A | ON161763 |
|  | *C. parvum* | ‒ | 1 | *ssu* rRNA | ‒ | ‒ | ‒ | ‒ |
| *Cryptosporidium* | *C. parvum* | IIaA15G2R1 | 10 | *gp*60 | MK099855 | 13‒853 | None | ON165665 |
|  | *C. parvum* | IIaA16G3R1 | 1 | *gp*60 | MK034693 | 55‒901 | C308A | ON165666 |
|  | *C. parvum* | IIaA17G1R1 | 1 | *gp*60 | KF147537 | 13‒828 | None | ON165667 |
|  | *C. parvum* | IIdA17G1R1 | 1 | *gp*60 | KY499053 | 15‒822 | None | ON165668 |
| *Blastocystis* sp. | ST1 | Allele 4 | 2 | *ssu* rRNA | AY618266 | 17‒602 | None | ON161764 |
|  | ST2 | Allele 12 | 1 | *ssu* rRNA | AB070987 | 11‒601 | G185A, T267A, A478C | ON161765 |
|  | ST4 | Allele 42 | 6 | *ssu* rRNA | AY244620 | 67‒564 | None | ON161766 |
| *E. bieneusi* | D | ‒ | 1 | ITS | AF101200 | 31-416 | None | ON167516 |
|  | Novel | ‒ | 1 | ITS | AF101199 | 31‒419 | C182T | ON167517 |

*bg*: β-giardin; Del: Deletion; *gdh*: Glutamate dehydrogenase; ITS: Internal transcribed spacer *gp*60: 60-kDa glycoprotein; *ssu* rRNA: Small subunit ribosomal RNA; *tpi*: Triose phosphate isomerase.
